# Fear of large carnivores causes a trophic cascade

**DOI:** 10.1038/ncomms10698

**Published:** 2016-02-23

**Authors:** Justin P. Suraci, Michael Clinchy, Lawrence M. Dill, Devin Roberts, Liana Y. Zanette

**Affiliations:** 1 Department of Biology, University of Victoria, Victoria, British Columbia, Canada V8W 2Y2; 2 Raincoast Conservation Foundation, Sidney, British Columbia, Canada V8L 3Y3; 3 Department of Biology, University of Western Ontario, London, Ontario, Canada N6A 5B7; 4 Department of Biological Sciences, Simon Fraser University, Burnaby, British Columbia, Canada V5A 1S6

## Abstract

The fear large carnivores inspire, independent of their direct killing of prey, may itself cause cascading effects down food webs potentially critical for conserving ecosystem function, particularly by affecting large herbivores and mesocarnivores. However, the evidence of this has been repeatedly challenged because it remains experimentally untested. Here we show that experimentally manipulating fear itself in free-living mesocarnivore (raccoon) populations using month-long playbacks of large carnivore vocalizations caused just such cascading effects, reducing mesocarnivore foraging to the benefit of the mesocarnivore's prey, which in turn affected a competitor and prey of the mesocarnivore's prey. We further report that by experimentally restoring the fear of large carnivores in our study system, where most large carnivores have been extirpated, we succeeded in reversing this mesocarnivore's impacts. We suggest that our results reinforce the need to conserve large carnivores given the significant “ecosystem service” the fear of them provides.

## Abstract (web summary)

Top predators may indirectly influence ecological processes through fear-induced behavioural changes in their prey. By experimentally manipulating this ‘landscape of fear', Suraci *et al*. show that fear of large carnivores in a mesopredator can cause cascading effects down the food web that benefit its prey.

## Graphical abstract

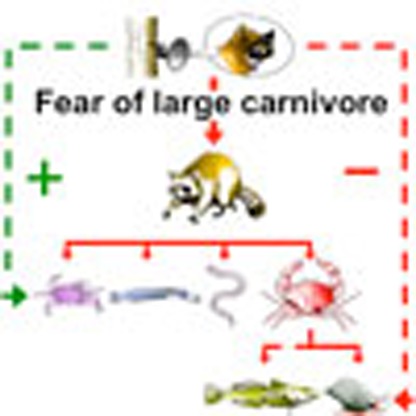

Large carnivores are fearsome predators that pose real and perceived threats to human life^1^ and livelihoods^1^,^2^,^3^,^4^,^5^, which is why humans have attempted, and largely succeeded, at extirpating them everywhere^5^,^6^,^7^. The loss of large carnivores is now being recognized as possibly ‘humankind's most pervasive influence on nature'^8^, in part because the fear (perceived predation risk^9^,^10^,^11^) they inspire in other animals may constitute a significant “ecosystem service” critical to conserving biodiversity and ecosystem function^6^,^12^,^13^,^14^. Being at the top of the food chain, large carnivores can play a dual role in structuring ecosystems by affecting both large herbivores and mesocarnivores, causing both “tri-trophic cascades” (large carnivore–herbivore–plant) and “mesopredator cascades” (large carnivore–mesopredator–mesopredator's prey), affecting diverse species at multiple lower trophic levels^6^,^8^,^12^,^13^,^14^. By both killing and frightening their prey, large carnivores could have a dual impact on these dual cascades, for the straightforward reason that frightened prey (in this case large herbivores and mesocarnivores) eat less^9^,^11^,^12^,^15^. The mere presence of large carnivores may therefore give rise to a “landscape of fear”^9^, buffering lower trophic levels from overconsumption by large herbivores and mesocarnivores. Failing to consider fear risks substantially underestimating the role large carnivores play, since fear may be as or more important than direct killing in causing trophic cascades, according to current theory and experiments primarily on captive invertebrates^16^,^17^,^18^,^19^. Given the potential for human-large carnivore conflict, there have been justifiable calls for direct experimental evidence that the fear large carnivores inspire can provide a significant “ecosystem service”^4^,^20^. The absence of such direct evidence to date is due to the challenge of experimentally manipulating fear in free-living wildlife, it being only very recently experimentally demonstrated that fear itself is powerful enough to affect wildlife population dynamics^21^.

To test whether the fear of large carnivores can itself cause cascading effects on mesocarnivore foraging and multiple lower trophic levels, we conducted a spatially and temporally replicated field experiment in which we manipulated fear using month-long playbacks of large carnivore vocalizations (Fig. 1; Supplementary Table 1). The experiment was conducted on wild, free-living raccoons (*Procyon lotor*) on several small coastal Gulf Islands (BC, Canada). The raccoon is a mesocarnivore subject to much research regarding “mesopredator release”^14^,^22^. Most of the large carnivores known to hunt (wolf (*Canis lupus*), cougar (*Puma concolor*)) or harass (black bear (*Ursus americanus*)) raccoons were extirpated from the Gulf Islands last century^22^,^23^, the sole remaining large carnivore being the domestic dog (*Canis lupus familiaris*), which harasses and kills raccoons here, and has been present for millennia, having always been kept by local aboriginal peoples^24^. In a previous mensurative experiment comparing Gulf Islands with and without raccoons^22^, we documented that raccoons impact multiple marine species, reducing the abundance of intertidal crabs and fish, and even subtidal red rock crabs (*Cancer productus*).

To experimentally test whether the fear of large carnivores could itself mediate the impacts of raccoons on marine biota, we broadcast large carnivore predator (dog) or non-predator (local pinnipeds; harbour seal (*Phoca vitulina*), Steller sea lion (*Eumetopias jubatus*)) vocalizations over two large sections of shoreline for 1 month, and then reversed the treatments for a second month, using a repeated measures design to spatially replicate our results, which we further spatially and temporally replicated by repeating the same manipulation on a different island the following year. We assayed the immediate reaction of raccoons to the large carnivore predator and non-predator vocalizations by directly observing their reactions to 10 s playbacks, and assessed their response to the month-long playbacks using multiple video surveillance and time-lapse cameras to continuously film both experimental sections of shoreline over both entire month-long playback periods

To test the cascading effects of our experiment on lower trophic levels, we utilized the same methods (intertidal quadrats and subtidal crab trapping) used in our previous mensurative experiment to evaluate effects on raccoon prey^22^, and we conducted fish trapping and a mark-recapture experiment additionally quantifying effects on species not directly eaten by raccoons, that are instead a competitor (staghorn sculpin (*Leptocottus armatus*)) and prey (periwinkle snail (*Littorina scutulata*)) of the prey (red rock crab) of raccoons^25^,^26^. Staghorn sculpins are subtidal fish that, like red rock crabs, enter the intertidal at high tide to feed on small invertebrates^25^, but unlike red rock crabs, evidently successfully escape being eaten by raccoons. Periwinkle snails are too small to be food for raccoons but are eaten by red rock crabs, which use a distinctive method of dispatching them, permitting the level of mortality to be quantified using standard mark-recapture procedures^26^.

Here we report significant cascading effects of the fear of large carnivores across multiple trophic levels in an intertidal food web, which effectively reversed the impacts of mesocarnivore populations on marine biota by markedly suppressing mesocarnivore foraging. These results indicate that the fear large carnivores inspire in their prey can account for a major component of their role in structuring ecosystems, reinforcing the value of large carnivore conservation in ensuring the continuation of this critical ecosystem service.

## Results

### Cascading effects of the fear of large carnivores

**Figure 1:**
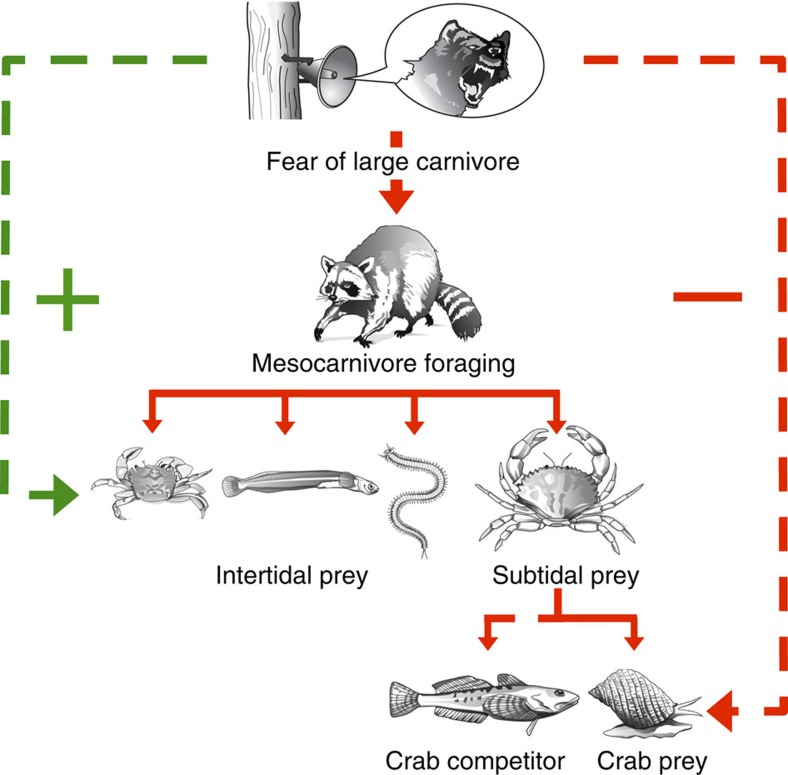
Fear of large carnivores caused a trophic cascade. Diagram illustrating how broadcasting playbacks of large carnivore vocalizations affected multiple lower trophic levels. Green and red arrows represent positive and negative effects, respectively, on foraging, abundance or survival. Solid arrows connect predator and prey; dashed arrows connect species affected, but not directly eaten, by another.

**Figure 2:**
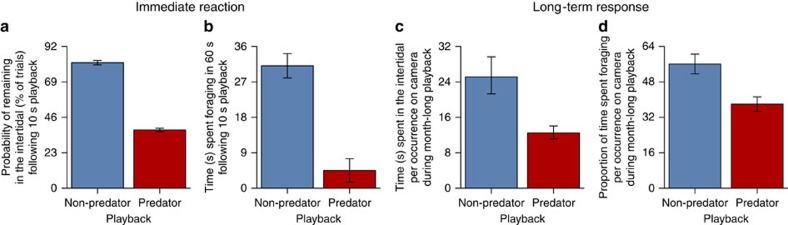
Fear of large carnivores reduced mesocarnivore foraging. (**a**) Probability of remaining in the intertidal (% of trials; log-linear test, *χ*^2^_1_=11.96, *P*<0.001; *n*=45 (predator) and 27 (non-predator)), and (**b**) time spent foraging (out of 60 s; ANOVA, *F*_1,33_=15.85, *P*<0.001; *n*=22 and 17) immediately following 10 s predator and non-predator playbacks. (**c**) Time spent in the intertidal (per occurrence on camera; Linear Mixed Effects Model (LMM), Likelihood Ratio (LR) *χ*^2^_1_=9.66, *P*=0.002; *n*=51 and 43), and (**d**) proportion of time spent foraging (per occurrence on camera; LMM, LR *χ*^2^_1_=11.86, *P*=0.001; *n*=62 and 52) during month-long predator and non-predator playbacks. Values are means±s.e.m.

**Figure 3:**
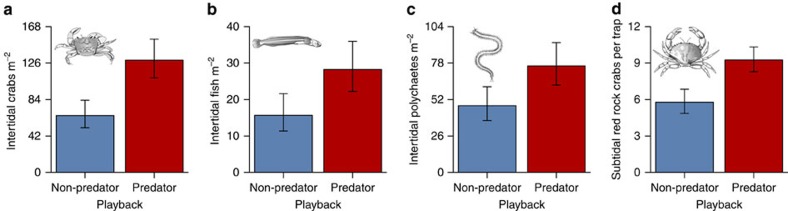
Fear of large carnivores benefited the mesocarnivore's prey. Abundance of (**a**) intertidal crabs (Quasi-Poisson Generalized Linear Model (GLM), *F*_1,36_=12.11, *P*=0.001; *n*=20 (predator) and 20 (non-predator)), (**b**) intertidal fish (Poisson GLM, Likelihood Ratio *χ*^2^_1_=5.15, *P*=0.023; *n*=20 and 20), (**c**) intertidal polychaete worms (Quasi-Poisson GLM, *F*_1,36_=4.54, *P*=0.039; *n*=20 and 20) and (**d**) subtidal red rock crabs (Poisson Generalized Linear Mixed Effects Model; Wald's *χ*^2^_1_=10.83, *P*=0.001; *n*=20 and 20) following month-long predator and non-predator playbacks. Values are means±s.e.m.

**Figure 4:**
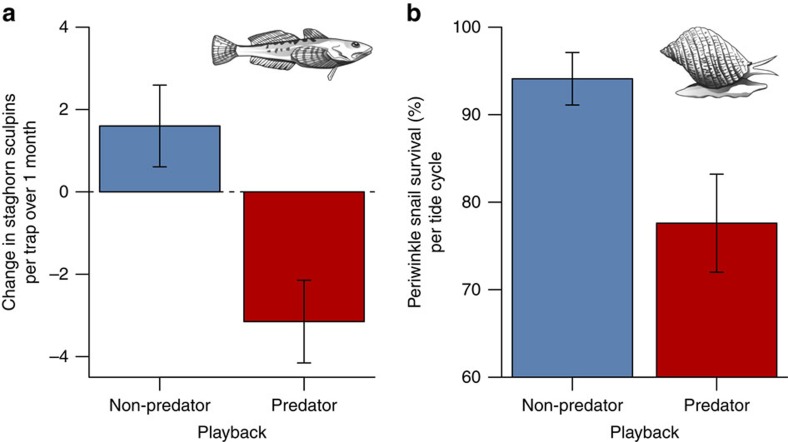
Fear of large carnivores affected a competitor and prey of the mesocarnivore's prey. (**a**) Change in abundance of staghorn sculpins over one month (Linear Mixed Effects Model, Likelihood Ratio *χ*^*2*^_*1*_=21.17, *P*<0.001; *n*=20 (predator) and 20 (non-predator)) and (**b**) survival of periwinkle snails per tide cycle (Binomial Generalized Linear Mixed Effects Model, Wald's *χ*^2^_1_=9.51, *P*=0.002; *n*=10 and 8) during month-long predator and non-predator playbacks. Values are means±s.e.m.

Fear of large carnivores dramatically reduced mesocarnivore foraging (Fig. 2; Supplementary Tables 2 and 3). The immediate reaction of raccoons to the 10 s predator playbacks was to either abandon foraging entirely by leaving the intertidal (Fig. 2a) or reduce foraging (Fig. 2b) and increase vigilance. Critically, these same responses persisted throughout the month-long playbacks. Large carnivore playbacks caused raccoons to spend less time in the intertidal (Fig. 2c), and less time feeding when they were present (Fig. 2d), with the cumulative consequence that they spent 66% less time foraging over the course of the month. This dramatic reduction in mesocarnivore foraging in turn dramatically benefitted the mesocarnivore's prey (Fig. 3; Supplementary Tables 4 and 5). Following the month-long large carnivore playbacks, there were 97% more intertidal crabs (Fig. 3a), 81% more intertidal fish (Fig. 3b), 59% more polychaete worms (Fig. 3c) and 61% more subtidal red rock crabs (Fig. 3d; Supplementary Fig. 1) compared with after the non-predator playbacks. Finally, fear of large carnivores clearly had cascading effects on multiple lower trophic levels (Fig. 1), as the reduction in raccoon foraging (Fig. 2c,d) that produced the increased relative abundance of red rock crabs (Fig. 3d) was associated with a decreased abundance of the red rock crab's competitor (staghorn sculpin; Fig. 4a) and led to lower survival of the red rock crab's prey (periwinkle snail; Fig. 4b; Supplementary Table 6).

## Discussion

Our results unambiguously experimentally demonstrate that the fear of large carnivores can itself cause a trophic cascade. Manipulating fear itself—by hanging a speaker from a tree—caused cascading effects on predation and competition at multiple trophic levels in the ocean (Fig. 1). Moreover, there were likely many more effects than we measured, potentially cascading down to the level of primary producers, since red rock crabs are themselves significant predators^26^ and periwinkle snails are significant grazers^27^ (Supplementary Discussion).

Our results additionally demonstrate that failing to consider the fear large carnivores inspire risks significantly underestimating their role, given the striking magnitude of the effects (Figs 3 and 4), and that these effects were comparable in magnitude to those documented in our previous mensurative experiment comparing islands with and without raccoons. The presence of raccoons on an island reduces the respective abundance of intertidal crabs and fish, and subtidal red rock crabs, by 90%, 94% and 38% (ref. 22), impacts all reversed by the 97%, 81% and 61% relative increase in each (Fig. 3) resulting from the reduction in raccoon foraging caused by the fear of large carnivores (Fig. 2). That the fear of large carnivores could itself be powerful enough to reverse these impacts corresponds with the fact that the human extirpation of most large carnivores from the Gulf Islands means raccoons here no longer have much to fear, and act accordingly, as they forage night and day (being more normally nocturnal) far from cover (deep into the intertidal)^22^, and rarely look up from eating, spending <1 s vigilant per minute (Methods). Our experiment reversed this now unrestrained foraging by restoring the fear of large carnivores to a system from which it has largely been lost, revealing the significance of the ecosystem service the presence of the now extirpated large carnivores (wolves, cougars and black bears) provided, solely through the fear they inspired.

Similar restorative effects of the fear of large carnivores have been attributed to the reestablishment of a “landscape of fear” accompanying the reintroduction or recolonization of large carnivores^6^,^9^,^13^,^28^,^29^; and broad-scale impacts accompanying the loss of large carnivores that cannot be explained by the reduction in direct killing alone have likewise been attributed to the associated loss of the fear of large carnivores^6^,^12^,^13^,^14^,^30^. The evidence to date that the fear of large carnivores can play a central role in structuring ecosystems comes largely from “natural experiments”^6^,^13^,^20^, and compelling alternative explanations often exist for the patterns observed^20^. Our results in no way refute these alternatives, but our being able to cause a trophic cascade by directly manipulating fear does conclusively demonstrate such a thing is possible, and so corroborates that the fear of large carnivores can play the role attributed to it.

Experimentally manipulating the fear of large carnivores demonstrably affected mesocarnivore behaviour (Fig. 2), which in turn evidently caused a fear cascade affecting the behaviour of at least some of the mesocarnivore's prey. Red rock crab abundance increased significantly over the course of the month-long large carnivore playbacks (Supplementary Fig. 1; Supplementary Discussion), which could only be due to a behavioural change in habitat use, as reproduction in this species requires at least a year^31^. Red rock crabs occur in large subtidal populations and move into the intertidal to forage, where they are killed and eaten by raccoons, which leave the remains, and thus chemical cues, of dead crabs in the water^22^. Surviving red rock crabs may modify their habitat use in response to these chemical cues, as has been demonstrated experimentally in other crab species^32^. The fear cascade evident from the increased abundance of red rock crabs may therefore have resulted from straightforward mechanisms: increasing the fearfulness of raccoons reduced their foraging (Fig. 2), which presumably led to reduced fearfulness in red rock crabs by decreasing chemical cues in the water, in turn leading to the red rock crabs' increased use of the intertidal (Supplementary Fig. 1).

The loss of large carnivores from habitats across the globe has been linked to far-reaching ecosystem-level consequences—including biodiversity loss and changes in habitat structure—caused by outbreaks of large herbivores and mesocarnivores^6^,^8^,^9^,^12^,^14^,^22^,^28^,^29^,^30^. Our results suggest that restoration of these ecosystems will require more than just addressing the overabundance of these middle trophic level species, for example, through hunting or removal programs. Such numerical suppression may only affect a subset of hyper-abundant large herbivore and mesocarnivore populations, while the remaining individuals are free to engage in unrestricted foraging. Effective ecological restoration may depend on re-establishing the fear of large carnivores in these ecosystems, which has the potential to affect entire populations of their prey (rather than just those individuals subject to direct killing or removal)^17^,^33^, suppressing prey foraging behaviour and thereby mitigating the impacts of overconsumption on lower trophic level species. The potential ecosystem-level benefit of the mere presence of large carnivores, and the ‘landscape of fear' they produce, should therefore be a central consideration in making informed management decisions regarding large carnivore populations.

Our experimental results support the contention that, when it comes to conserving biodiversity and maintaining healthy ecosystems, fear has its uses^6^,^12^,^13^,^14^. By inspiring fear, the very existence of large carnivores on the landscape, in and of itself, can provide a critical ecosystem service human actions cannot fully replace, making it essential to maintain or restore large carnivores for conservation purposes on this basis alone^6^,^12^,^14^,^30^. Ensuring the continuation of this critical ecosystem service the fear of large carnivores provides requires attenuating our own fear of them, which can be accomplished by promoting tolerance and coexistence with large carnivores as an accompaniment to other programs to reduce human-large carnivore conflicts^1^,^2^,^3^,^4^,^5^,^6^,^34^,^35^.

## Methods

### Study area

Vegetation in the Gulf Islands falls within the Coastal Douglas Fir (*Psuedostuga menziesii*) biogeoclimatic zone, and the region experiences a mild Mediterranean climate^36^. This work was conducted on four Gulf Islands. Coal Island (140 ha; 48° 41′ 03′′ N, 123° 22′ 32′′ W) is a single-owner private island consisting of ∼78% forest. Portland Island (225 ha; 48° 43′ 33′′ N, 123° 22′ 20′′ W) and Wallace Island (87 ha; 48° 56′ 34′′ N, 123° 33′ 04′′ W) are both fully forested parkland, being entirely within the Gulf Islands National Park Reserve (Portland), or mostly BC Provincial Park (Wallace, 83% of land area). Penelakut Island (954 ha; 48° 57′ 30′′ N, 123° 38′ 34′′ W) is the traditional territory of the Penelakut First Nation and home to ∼350 people, all residing in a small village on the north end of the island. The majority of Penelakut Island (∼86%) is forested. Domestic dogs were present on all Gulf Islands on which this study was conducted, either as the pets of permanent residents (Coal and Penelakut Islands) or accompanying park visitors (Portland and Wallace Islands), though study sites were chosen well away from areas of high human and dog use to minimize interference.

### Motivation and objectives

We experimentally manipulated the fear of large carnivores over 2 years and at multiple sites in the Gulf Islands, achieving both temporal and spatial replication of our results. In 2013, we tested the immediate reaction of raccoons to 10 s playbacks of large carnivore vocalizations on Coal, Portland and Wallace Islands. We then used month-long playbacks of large carnivore vocalizations to test for long-term behavioural responses by raccoons and cascading effects on marine biota, on Coal Island in 2013 and on Penelakut Island in 2014. The objectives in these 2 years were similar but complementary. In 2013, we focused on testing whether the fear of large carnivores was sufficient to mitigate the impacts of raccoons on intertidal and shallow subtidal prey. In 2014, we sought (1) to replicate the results from the Coal Island experiment concerning raccoon prey abundance, while expanding the focus of the study, (2) to test whether the effects of fear on raccoon behaviour observed in 10 s playback experiments persisted throughout our month-long treatments and (3) to test for further cascading effects of fear among intertidal and shallow subtidal species not directly eaten by raccoons. All work was conducted in compliance with the guidelines of the Canadian Council on Animal Care, and was approved by the Animal Care and Use Committees of the University of Victoria and the University of Western Ontario.

### Preparing the playbacks

We manipulated the fear of large carnivores using playbacks of domestic dog (large carnivore predator) and local pinniped (harbour seal and Steller sea lion; non-predator) vocalizations. Pinniped vocalizations provide an excellent control for dogs; the two call types are qualitatively similar, and Gulf Islands raccoons are certain to be as familiar with pinniped as with dog vocalizations. Most importantly, pinnipeds represent no threat to raccoons, and analyses of red rock crab abundance data verified that there was no difference between pinniped playbacks and silence with respect to raccoon impacts on marine prey (Supplementary Discussion). Sound files were acquired from online audio and video databases, and library archives. In testing the immediate reaction of raccoons to large carnivore vocalizations, we used multiple 10 s exemplars of predator and non-predator vocalizations (10 dog and 5 pinniped), and matched the temporal properties (duration, attack and number of staccato elements) of these two groups of playbacks by visually inspecting the spectrograms and waveforms of all exemplars^37^. We ensured that there were no differences in overall frequency characteristics between the two groups using *t*-tests to compare each of four frequency characteristics (peak: *t*_1,13_=−0.36, *P*=0.735; minimum: *t*_1,13_=1.46, *P*=0.180; maximum: *t*_1,13_=0.63, *P*=0.551; range: *t*_1,13_=0.62, *P*=0.556; *n*=10 (predator) and 5 (non-predator) for all tests). We broadcast 10 s calls at a mean (±s.d.) volume of 78.0 (±2.1) dB at 1 m, with no difference in volume between predator and non-predator treatments (*t*_1,13_=−1.3, *P*=0.234; *n*=10 and 5). All playbacks were broadcast using identical speakers (Nexxtech Mini Cube 2.0) and mp3 players (Coby Electronics MP301).

To test the long-term response of both raccoons and the nearshore marine community to the fear of large carnivores, we again used playbacks of dog (predator) and pinniped (non-predator) vocalizations. We composed playlists using multiple exemplars of both call types (*n*=11 predator and 9 non-predator exemplars) ranging in duration from 8 to 79 s, with no difference in duration between the two treatments (predator (mean±s.d.): 34.1±20.6 s; non-predator: 26.8±18.3 s; *t*_1,18_=0.84, *P*=0.412). These two sets of playbacks were again matched for temporal properties using visual inspection of spectrograms and waveforms, and we used *t*-tests to confirm that there were no differences in overall frequency characteristics between predator and non-predator playlists (peak frequency: *t*_1,18_=−0.03, *P*=0.973; minimum: *t*_1,18_=−1.44, *P*=0.180; maximum: *t*_1,18_=0.76, *P*=0.459; range: *t*_1,18_=0.86, *P*=0.400; *n*=11 (predator) and 9 (non-predator) for all tests). All calls were broadcast at a mean (±s.d.) volume of 86.1 (±2.9) dB at 1 m, with no difference in volume between predator and non-predator treatments (*t*_1,18_=−0.13, *P*=0.895; *n*=11 and 9). All playbacks were broadcast using identical speakers (Nexxtech Mini Cube 2.0) and mp3 players (The Source HeadRush 2GB mp3 player).

### Raccoon immediate reaction to large carnivore vocalizations

All 10 s playback trials were conducted by two researchers (J.P.S. and D.R.) between 15 May and 16 September 2013. We located diurnally active raccoons foraging in the intertidal and broadcast a randomly selected predator or non-predator playback from a concealed location. The raccoon's behaviour was video recorded immediately before and immediately following the 10 s playback using a handheld digital video camera with a × 70 optical zoom (Sony DCR-SX45 Handycam). Immediately following each trial, the distance between the speaker and the focal animal's location at the time of the playback was measured using a rangefinder (Bushnell Sport 450). Calls were broadcast at an average (±s.d.) distance of 35 (±16) m, and the distance between the speaker and the focal animal did not differ between predator and non-predator treatments (one-way analysis of variance (ANOVA); *F*_1,70_=0.96, *P*=0.33; *n*=45 (predator) and 27 (non-predator)). Habitat variables that could potentially impact the raccoon's ability to hear the playback—including wind speed, rainfall and wave action—were measured for each trial, and showed no difference between treatments (Wilcoxon rank-sum test; 0.18>*P*>0.87 for all variables; *n*=45 and 27 for all tests). As the reaction of conspecifics to our playback treatments could conceivably have influenced the focal animal's behaviour, we also quantified the number of conspecifics within 50 m of the focal animal at the time of the playback. In general, raccoons were >50 m from any conspecific during playback trials (median (range) conspecifics within 50 m=0 (0, 5)), and there was no difference in the number of conspecifics present between the two treatments (Wilcoxon rank-sum test, *W*=687, *P*=0.27, *n*=45 and 27). To minimize the likelihood of repeated sampling of raccoons, playback locations on a given island were separated by a median linear distance of 266 m, comparable to the spacing of sampling points (273 m) commonly utilized in raccoon live-trapping studies^38^. The minimum distance between trials of the same treatment did not differ between predator and non-predator treatments (ANOVA; *P*=0.17; *n*=45 (predator) and 27 (non-predator)). Each playback type was only played once at a given location.

A single researcher (D.R.), who was blind to the playback treatment, scored all video recordings of 10 s playback trials, and estimated three behavioural measures. First, we scored whether or not the focal raccoon fled the intertidal (that is, moved from the exposed shoreline into the surrounding forest) within the 3 min immediately following the playback. For those individuals that did not flee, we calculated the amount of time (s) devoted to foraging and to vigilance (see Supplementary Fig. 2 for behaviour scoring methodology) in the 60 s immediately before and immediately following the playback. Gulf Islands raccoons spent <1 s of every minute vigilant in the 60 s before the playback (0.9±0.3 s (mean±s.e.m.), range=0–14), and instead spent the great majority of their time foraging. We calculated the change in foraging as the time spent foraging following the playback minus time spent foraging before the playback. An identical calculation was made for the change in vigilance. We report that raccoons significantly decreased foraging in response to the predator playback, relative to the non-predator playback. We found a correspondingly strong increase in vigilance in the 60 s following the predator playback (vigilance increased by 27.1±1.7 (s.e.m.) s, relative to pre-playback, *n*=22 raccoons), as compared with the non-predator playback (vigilance increased by 9.7±1.7 s, *n*=17; Supplementary Table 2).

### Raccoon long-term response to the fear of large carnivores

The month-long playback manipulations were conducted on Coal Island in 2013 (10 May to 13 July) and Penelakut Island in 2014 (28 June to 25 August). On each island, we chose two shoreline sites separated by sufficient distance (1.2 km on Coal and 2.7 km on Penelakut) that sounds were not detectable between sites. We used a repeated measures design, presenting both predator and non-predator treatments at all sites and alternating the order of treatment presentation between sites on each island, allowing us to control for the effects of site and seasonality on raccoon behaviour and intertidal community data. On a given island, each site received either the predator or non-predator treatment for 28 days (treatment period 1), followed by the opposite treatment for a subsequent 28 days (treatment period 2), with <1 week separating the two treatment periods. Starting treatment (predator or non-predator) was randomly assigned. Throughout each treatment period, we monitored raccoon behaviour (Penelakut), the abundance of intertidal prey (Coal) and subtidal prey (Coal and Penelakut), and the survival and abundance of marine species not directly eaten by raccoons (Penelakut).

At all sites, five identical sets of speakers and mp3 players housed in weatherproof boxes were deployed at regular intervals along sections of shoreline, attached to trees just above the high water line. Speakers played multiple exemplars of large carnivore predator (dog) or non-predator control (pinniped) vocalizations at regular intervals throughout each 28-day treatment period. On Coal Island in 2013, the speaker systems were deployed at 25-m intervals across 100 m of shoreline, and broadcast either predator or non-predator playbacks 24 h per day (raccoons on Coal were active both day and night; J.P.S., pers. obs.). Each speaker played a randomized playlist of calls interspersed by periods of silence, broadcasting calls 40% of the time and remaining silent for 60% of the time. To reduce the likelihood of raccoon habituation to the playbacks, speakers were intermittently turned off completely for 1–4 days at a time during each 28-day treatment period^21^ such that speakers were active for a total of 19 days during treatment period 1 and 18 days during treatment period 2.

On Penelakut Island in 2014, the five speaker systems were deployed at 50-m intervals across 200 m of shoreline and broadcast calls only at night (1900 to 0900 hours), as this is the period when raccoons on Penelakut are most active (J.P.S., personal observation). Speakers remained on for the full 28 days of each treatment period and played randomized playlists of either dog or pinniped calls 20% of the time, remaining silent for 80% of the time. In addition, two motion-sensitive speaker systems were deployed at each site, one at each end of the 200-m treatment area. These custom-built speaker systems used identical components to those described above, but were modified to incorporate a motion sensor, which activated a 10 s playback (using the same sets of calls described above) when triggered by a raccoon passing within ∼5 m of the sensor. These speaker systems were always active, but remained silent unless triggered. There is no evidence that the minor methodological differences in fear manipulation between the 2 years of the experiment had any effect on our results. Indeed, the effect size on subtidal red rock crab abundance was identical in both years (Supplementary Discussion).

We measured the long-term effects of the fear of large carnivores on several aspects of raccoon foraging on Penelakut Island in 2014, using a network of cameras deployed at each treatment site. Two colour/infrared video surveillance cameras (Speco Technologies HT7915DNV Bullet Cameras), recording to custom-built digital video recording systems^21^, were deployed within each treatment site and spaced 100 m apart. We estimated the duration of time raccoons spent in the intertidal for all independent raccoon occurrences on camera as the time (s) from an individual raccoon first entering a camera's field of view to when it exited the field of view. Two time-lapse cameras (Moultrie Game Spy M-990i trail cameras set to ‘time-lapse' mode) spaced 100 m apart were deployed within each treatment site and programmed to record one photograph every 30 s. For each time-lapse image in which a raccoon's entire body was visible, we scored the raccoon's behaviour as either foraging or vigilant (Supplementary Fig. 2). For each individual raccoon, we determined the total number of photos in which it appeared, and calculated the proportion of those photos scored as foraging. A single individual (J.P.S.) scored all images of raccoons taken from time-lapse cameras. To determine whether these scores were repeatable, five independent observers, blind to the fear manipulation treatment, re-scored a total of 184 time-lapse photos (21% of all photos). Agreement between the original scores (used in the analyses presented here) and those made by the five observers was 80%.

For both camera types (video and time lapse), we only used data from raccoons recorded between 1900 and 0900 hours, when playback systems on Penelakut Island were active. Unless multiple raccoons occurring on a single camera could be classified with certainty as unique individuals, raccoon occurrences on camera were only treated as independent if they were separated by >30 min (refs 39, 40). All raccoon behaviour variables were calculated per camera per night of the treatment period^40^,^41^; ‘camera night' is therefore the unit of replication in all behavioural analyses (see below). Camera placement remained constant throughout the duration of the experiment, and we therefore analysed all behavioural data using mixed effects models with Camera ID included as a random effect to account for variation due to camera placement.

### Measuring cascading effects of fear

In 2013, we used standard quadrat sampling methods to test whether our month-long playback manipulations affected the abundance of raccoon intertidal prey. On the basis of previous work^22^ and direct observations of raccoon foraging, our *a priori* prediction was that our fear manipulations would affect the abundances of small (<5 cm carapace width) intertidal crabs (shore crabs (*Hemigrapsus oregonensis*, *H. nudus*), black-clawed crabs (*Lophopanopeus bellus*), porcelain crabs (*Petrolisthes* spp.) and juvenile northern kelp crabs (*Pugettia producta*)), intertidal fish (pricklebacks (family Stichaeidae) and northern clingfish (*Gobiesox maeandricus*)), and polychaete worms (families Terebellidae, Orbiniidae, Nereidae and Glyceridae). Following methods described in Suraci *et al*.^22^, we quantified species abundance in ten 0.25 × 0.25-m quadrats at each treatment site on Coal Island at the end of each 28-day treatment period.

To test whether our playback manipulations affected red rock crab abundance, we trapped crabs across 200-m sections of shoreline centred at each treatment site by setting five collapsible mesh crab traps per site just below the low intertidal zone, spaced 50 m apart and left in place for 24 h (ref. 22). On Coal Island in 2013, crab traps were set at the end of each month-long treatment period. Sampling effort was intensified on Penelakut Island in 2014 such that crab trapping was conducted once before the application of any playback treatments (on 11 June 2014) to establish a pretreatment baseline crab abundance, and then at the end of each week during both month-long treatment periods (that is, four times per treatment period). In both years, trap locations remained constant across all trapping sessions at a given site.

The effects of the fear of large carniovres may extend beyond those species directly subject to raccoon predation to affect the competitors and prey of the raccoons' prey. Red rock crabs are major intertidal predators, and may compete for resources with other intertidal predators of similar body size, including staghorn sculpins, which are not subject to raccoon predation (Supplementary Discussion). We tested whether our fear manipulation treatments affected staghorn sculpin abundance by setting conical fish traps (minnow traps) across both treatment sites on Penelakut Island in 2014. Five traps, spaced 50 m apart and baited with ∼100 g of frozen herring, were set in the mid intertidal zone at each site and left in place for 24 h. Traps were deployed three times per 28-day treatment period, once immediately before the start of each treatment period and again at the mid-point and end of each treatment period. The same trap locations were used for all fish trap deployments at both sites.

Red rock crabs are known to affect the abundance of several species of gastropod prey, including periwinkle snails (Supplementary Discussion). We hypothesized that, during predator treatments, reduced raccoon predation on red rock crabs would result in increased red rock crab predation on periwinkle snails relative to non-predator treatments. To test this, we performed four replicate short-term snail mark-recapture experiments nested within our fear manipulation experiment on Penelakut Island in 2014, comparing the proportion of marked snails killed by red rock crabs during predator and non-predator treatments. Periwinkle snails were collected from high intertidal beds of *Fucus* algae at each site and transported back to the laboratory where they were marked with a small dab of acrylic paint on the apex of the shell, and held overnight in seawater tanks. Following Rochette and Dill^42^, snails were released the next day at each of four release points (spaced 30 to 50 m apart) within the 200-m treatment area at each site. All release points were located at the same tide level (1.0 m above mean lower low water) on areas of flat rock away from large boulders or crevices^42^. Twenty snails were released within a 5-cm radius of each release point during afternoon rising tides when release points were submerged under at least 1.5 m of water. The following morning at low tide, immediately following exposure of the release points, two researchers searched a 4-m radius around each point, recovering marked live snails and the marked apices of crushed snail shells. Red rock crab predation on snails produces a characteristic shell crushing pattern^26^, allowing one to reliably diagnose snail mortality due to red rock crab predation, and counting only shell apices rather than all crushed shell fragments ensures that each crushed snail is only counted once^42^. We estimated the proportion of marked snails surviving red rock crab predation over one tide cycle as the number of live snails recovered divided by the total number of live snails and crushed apices recovered at each release point (the fate of snails not recovered could not be reliably ascribed to red rock crab predation). To minimize potential bias due to low recovery rates, we only used data from trials for which at least 50% of the 20 released snails were recovered, alive or dead (*n*=18 trials). For these trials, the average recovery rate was 71% (range=50–95%), and did not differ between predator and non-predator treatments (*t*_1,16_=−0.04, *P*=0.97). This snail mark-recapture study was replicated four times on Penelakut Island in 2014, at the mid-point and end of each month-long treatment period, using the same four release points at each site throughout both treatment periods.

### Statistical analyses

All model assumptions were checked using statistical tests for normality and homogeneity of variance, and the fit of all models was visually inspected using residual versus fitted value plots and quantile–quantile plots^43^. Where appropriate, means and s.e.'s were calculated on normalized data, and back-transformed to the original scale of the data for presentation in figures. All (Generalized) Linear Mixed Effects Models (LMM) were fit using the ‘lme4' package in R^44^.

We used a log-linear analysis to test whether playback treatment affected the proportion of trials in which the focal raccoon fled the intertidal following 10 s playbacks, using a model that included terms for treatment, island and a treatment × island interaction. Data on both the change in foraging and the change in vigilance exhibited by raccoons that did not flee the intertidal following 10 s playbacks were Box–Cox transformed and analysed using separate two-way ANOVA models including the main effects of treatment and island, and a treatment × island interaction (Supplementary Table 2).

We estimated the duration of time spent in the intertidal during month-long playback treatments for all independent raccoon occurrences on video surveillance cameras, and then used the median duration per camera night in our analysis. Median duration data were natural log-transformed and analysed using a LMM (Supplementary Table 3). We estimated the proportion of time that raccoons spent foraging when present in the intertidal from time-lapse camera data, as described above, and then calculated the average of these ‘proportion foraging' values for all individuals on a given camera night, weighted by the total number of photos of each individual. Raccoon occurrences on camera that produced fewer photos were thereby devalued relative to occurrences with many photos and thus more information. Nightly weighted mean proportions of time spent foraging were then analysed using LMM (Supplementary Table 3). The significance of model terms was tested using Likelihood Ratio Tests^43^. In both behavioural analyses, we tested for main effects of treatment and study site as well as a treatment × site interaction. We also tested for a main effect of time since the start of the treatment period (‘night', measured in days: 1–28) and an interaction between treatment and night. This allowed us to determine whether raccoon behavioural responses to the treatments changed over the course of the treatment period (for example, due to habituation) and whether any such changes differed between predator and non-predator treatments. Finally, we tested for a three-way treatment × site × night interaction. We found no evidence for raccoon habituation to predator playbacks across the month-long treatment periods in either behavioural measure; the effect of night and the treatment × night interaction were nonsignificant in both analyses (Supplementary Table 3).

Quadrat data on the abundances of intertidal prey were analysed using Generalized Linear Models with a Poisson distribution. All models were checked for overdispersion^43^, and those showing evidence of overdispersion (intertidal crabs and polychaete worms) were refit using the Quasi-Poisson distribution. Treatment, site and their interaction were included as fixed effects in all Generalized Linear Models (Supplementary Table 4). Following Zuur *et al*.^43^, the significance of all main effects and interactions was tested using Likelihood Ratio Tests for models fit with the Poisson distribution (intertidal fish (Fig. 3b)), and F-tests for models fit with the Quasi-Poisson distribution (intertidal crabs (Fig. 3a) and polychaete worms (Fig. 3c)).

We analysed the effect of the fear manipulation treatments on shallow subtidal red rock crab abundance across the 2 years of the experiment, using as our response variable the number of red rock crabs caught per trap at the end of treatment periods 1 and 2 in both 2013 and 2014. These data were analysed using a Generalized Linear Mixed Effects Model (GLMM) with a Poisson distribution and checked for overdispersion (ratio of null to residual deviance=1.17). We tested for main effects of treatment and year, and their interaction. Within a given year, sites and trap locations remained constant across treatment periods, so site and trap location were included in the analysis as nested random effects (trap location nested within site). The significance of main effects and interactions in the GLMM was tested using type II Wald's *χ*^2^-test^45^ (Supplementary Table 5).

We calculated the change in staghorn sculpin abundance across each treatment period by subtracting the number of sculpins caught in each trap at the mid-point and end of each treatment period from the number caught in the same trap immediately before that treatment period. These data were analysed using LMM, including trap location (constant across sampling events) as a random effect. We tested for the main effects of treatment and site, and a treatment × site interaction (Supplementary Table 6).

The effect of our playback treatments on the proportion of marked periwinkle snails escaping crab predation was determined by scoring all surviving snails as 1 and all crushed snails as 0 in each trial of the mark-recapture experiment, and analysing these data using GLMM with a binomial error distribution. There was no evidence for overdispersion in these data (ratio of null to residual deviance=1.10). Snail release point (constant across all trials at a given site) was included as a random effect. We again tested for the main effects of treatment and site, and a treatment × site interaction (Supplementary Table 6).

## Additional information

**How to cite this article:** Suraci, J. P. *et al*. Fear of large carnivores causes a trophic cascade. *Nat. Commun.* 7:10698 doi: 10.1038/ncomms10698 (2016).

## Supplementary Material

Supplementary Information Supplementary Figures 1-2, Supplementary Tables 1-6, Supplementary Discussion and Supplementary References
