# Supplementary material for: Fear of large carnivores causes a trophic cascade
Source: Nat Commun. 2016 Feb 23;7:10698. doi: 10.1038/ncomms10698 (PMC4766389; doi:10.1038/ncomms10698)
Supplement: Supplementary Information — Supplementary Figures 1-2, Supplementary Tables 1-6, Supplementary Discussion and Supplementary References [file ncomms10698-s1.pdf]

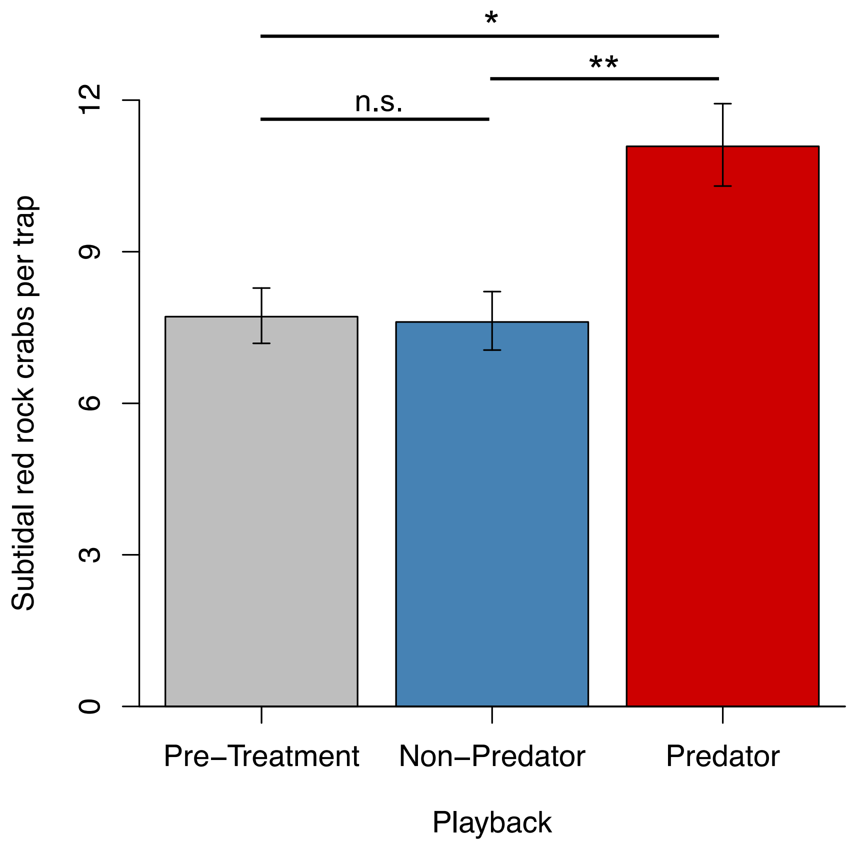

Supplementary Figure 1 | Fear of large carnivores affects red rock crab abundance. Red rock crab abundance compared between the pre-treatment, predator and non-predator playback periods sampled in the course of the more intensive red rock crab sampling conducted in 2014. Red rock crabs were trapped once prior to the start of any playback treatments (pre-treatment, n = 10), and then weekly during month-long predator (n = 40) and non-predator (n = 40) treatments (Linear Mixed Effects Model with Tukey's Post-Hoc Test; n.s. = not significant, *$P < 0.05$, **$P < 0.001$). Values are means ± s.e.m. For further details on this analysis, see Supplementary Discussion and Supplementary Table 5.

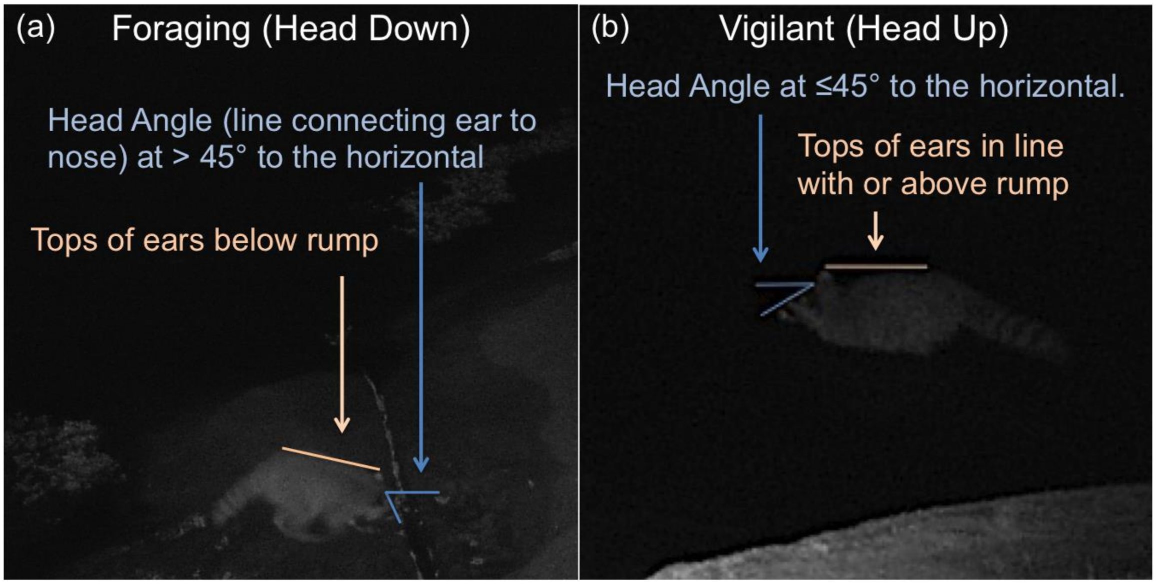

Supplementary Figure 2 | Raccoon behaviour scoring. Examples of the protocol used to score time spent foraging or vigilant based on raccoon head position in video recordings of 10 s playback trials (see Fig. 2B) and in 30 s time-lapse photos from month-long playback manipulations (see Fig. 2D). Videos (10 s playbacks) or photos (month-long playbacks) were scored as: (**a**) foraging if the head angle (angle between a line connecting the ears and nose of a raccoon head in profile and the horizontal) was > 45° *or* if the tops of the ears were below the rump; or (**b**) vigilant if the head angle was ≤ 45° *and* the tops of the ears were in line with or above the rump.

Supplementary Table 1 | Overview of methods used in this study to test hypotheses and specific predictions

Hypothesis	Prediction	Methods
(1) Raccoons reduce intertidal foraging in response to perceived predation risk from large carnivores	(i) Raccoons will react to immediate risk of predation by either abandoning intertidal foraging altogether or reducing foraging in favour of vigilance	10 s playbacks of predator or non-predator vocalizations to free-living raccoons foraging in the intertidal across multiple Gulf Islands. Video recording behaviour immediately prior to and immediately following playback
	(ii) Raccoons will reduce time allocation to intertidal foraging as a long-term response to high perceived risk of predation by large carnivores	Month-long playbacks of predator or non-predator vocalizations presented over sections of shoreline across two Gulf Islands. Raccoon behaviour continuously monitored by surveillance video and time-lapse cameras
(2) By reducing raccoon foraging, fear of large carnivores indirectly benefits raccoon intertidal and shallow subtidal prey	Abundance of intertidal vertebrates and invertebrates and shallow subtidal red rock crabs will be higher following month-long predator treatments, relative to non-predator treatments	- Intertidal quadrat sampling to monitor abundance of intertidal crabs, fish and polychaete worms<br>- Shallow subtidal crab trapping to monitor red rock crab abundance
(3) By benefiting raccoon prey, effects of large carnivore-induced fear will further cascade to negatively affect species not directly eaten by raccoons	(i) Abundance of staghorn sculpins (red rock crab competitors) will decrease during predator treatments	Intertidal fish trapping of staghorn sculpins throughout month-long fear manipulations
	(ii) Increased abundance of red rock crabs will lead to increased predation on periwinkle snails	Snail mark-recapture experiments nested within month-long fear manipulations, quantifying the proportion of snails surviving crab predation over a single tide cycle

Supplementary Table 2 | Model results for raccoon immediate reactions to 10 s playbacks

Results from Log Linear (probability of remaining in the intertidal) and Two-way ANOVA (change in time spent foraging and change in vigilance) models for raccoon immediate reactions to 10 s playbacks. Results presented here are the full model output from analyses illustrated in Fig. 2A (probability of remaining in the intertidal) and Fig. 2B (change in time spent foraging), along with the full results of the change in vigilance analysis, presented in the main text.

	Probability of remaining in the intertidal		Change in time spent foraging		Change in vigilance	
	χ^2	P-value	F-value	P-value	F-value	P-value
Treatment	11.957	***<0.001***	15.848	***<0.001***	11.761	***0.002***
Island	0.270	0.873	0.135	0.874	4.126	***0.025***
Treatment x Island	1.530	0.465	1.648	0.208	1.399	0.261

Supplementary Table 3 | Model results for raccoon responses to month-long playback manipulations

Results from Linear Mixed Effects Models for raccoon long-term behavioural responses to month-long predator and non-predator treatments. Results presented here are the full model output from analyses illustrated in Fig. 2C (time spent in the intertidal) and Fig. 2D (proportion of time spent foraging).

	Time spent in intertidal		Proportion of time spent foraging	
	LRT[1] χ^2	P-value	LRT χ^2	P-value
Treatment	9.661	***0.002***	11.285	***0.001***
Site	0.822	0.365	0.156	0.693
Night	0.096	0.870	2.563	0.109
Treatment x Site	0.027	0.346	2.626	0.105
Treatment x Night	0.462	0.497	1.654	0.198
Site x Night	1.698	0.193	0.092	0.762
Treatment x Site x Night	2.435	0.119	3.322	0.068

[1]Likelihood Ratio Test

Supplementary Table 4 | Model results for the effects of month-long playback manipulations on raccoon intertidal prey

Results from Generalized Linear Models testing the effect of month-long predator and non-predator treatments on raccoon intertidal prey. Results presented here are the full model output from analyses illustrated in Fig. 3A (intertidal crabs m^{-2}), Fig. 3B (intertidal fish m^{-2}) and Fig. 3C (polychaete worms m^{-2}).

	Intertidal crabs		Intertidal fish		Polychaete worms	
	F-value	P-value	LRT[1] χ^2	P-value	F-value	P-value
Treatment	12.110	***0.001***	5.148	***0.023***	4.541	***0.039***
Site	2.369	0.133	3.994	***0.046***	1.686	0.202
Treatment x Site	0.544	0.465	0.131	0.717	0.003	0.955

[1]Likelihood Ratio Test

Supplementary Table 5 | Model results for the effects of month-long playback manipulations on red rock crabs

Results from (a) Generalized Linear Mixed Effects Model, (b) Linear Mixed Effects Model and (c) Tukey's Post-Hoc Test describing the indirect effect of month-long predator and non-predator treatments on shallow subtidal red rock crab abundance. The full model output from the analysis illustrated in Fig. 3D (subtidal red rock crabs per trap) is presented in (a), while (b) and (c) are corroborative analyses, the results of which are illustrated in Supplementary Fig. 1.

(a) 2013 and 2014 data pooled

	Wald's χ^2	P-value
Treatment[1]	10.828	***0.001***
Year	2.814	0.093
Treatment x Year	0.577	0.448

(b) 2014 data only

	LRT[1] χ^2	P-value
Treatment[1]	17.365	***<0.001***
Site	4.778	***0.029***
Treatment x Site	3.973	0.137

[1]Likelihood Ratio Test

(c) Tukey's Pairwise Post-Hoc Test comparing treatments for 2014 data

	Z-value	P-value
Pre-Treatment vs. Non-Predator	0.095	0.995
Pre-Treatment vs. Predator	2.532	***0.029***
Predator vs. Non-Predator	4.154	***<0.001***

Supplementary Table 6 | Model results for the effects of month-long playback manipulations on species not directly eaten by raccoons

Results from (Generalized) Linear Mixed Effects Models testing the cascading effects of month-long predator and non-predator treatments on intertidal and subtidal species not directly eaten by raccoons. Results presented here are the full model output from analyses illustrated in Fig. 4A (change in staghorn sculpins per trap) and Fig. 4B (periwinkle snail survival).

	Change in staghorn sculpins per trap		Periwinkle snail survival	
	LRT[1] χ^2	P-value	Wald's χ^2	P-value
Treatment	21.174	***<0.001***	9.508	***0.002***
Site	6.812	***0.009***	2.921	0.087
Treatment x Site	2.690	0.101	1.224	0.268

[1]Likelihood Ratio Test

Supplementary Discussion

Consistency of fear effects on red rock crab abundance across islands and years
We found a remarkable degree of repeatability in our experimental results, as the differences in crab abundance between predator and non-predator treatments were identical in 2013 and 2014. There were on average 2.9 more red rock crabs per trap following predator treatments than following non-predator treatments in each year (s.e.m: ± 2.2 [2013] and ± 1.5 [2014]). This finding highlights not only the repeatability of our methods, but also the consistency of the community-level effects of the fear of large carnivores across islands and years, in the Gulf Islands.

Comparing red rock crab abundance during treatments to pre-treatment baseline
The above analysis, as well as those presented in the main text, required using a subset of the data on crab abundance collected in 2014 (in which crab abundance was sampled prior to the experiment and weekly during each treatment period; see Methods in main text), to match data collection in 2013 (when crab abundance was sampled only during the last week of each treatment period). We subsequently analyzed the full 2014 dataset separately, allowing us to compare the abundance of red rock crabs measured across the two treatment periods with baseline levels of crab abundance measured prior to the application of any playback treatment (see Methods). The response variable for this analysis was the number of red rock crabs per trap, and we tested for the main effects of treatment (a three level factor: "pre-treatment", "non-predator" and "predator") and site, as well as a treatment x site interaction. Within each site, trap locations remained constant throughout all pre-treatment and treatment trapping sessions, and trap location was therefore included in the analysis as a random effect. Due to overdispersion in the dataset, we were unable to achieve adequate model fit using a Generalized Linear Mixed Effects Model with a Poisson distribution. We therefore natural log + 1 transformed the response variable and analyzed the data using a Linear Mixed Effects Model. Statistical tests for normality and homogeneity of variance, as well as visual inspection of residual vs. fitted value plots and quantile-quantile plots confirmed the adherence of the transformed data to all model assumptions. We found a significant overall effect of treatment on the number of red rock crabs per trap (Supplementary Table 5, Supplementary Fig. 1), and subsequently performed a Tukey's pairwise comparison *post hoc* test to test for significant differences between each combination of treatment levels (Supplementary Table 5). The predator playback treatment led to a significant increase in crab abundance relative to both the non-predator treatment and the pre-treatment baseline. However, crab abundance did not differ between the pre-treatment baseline and the non-predator treatment (Supplementary Fig. 1, Supplementary Table 5), confirming the validity of our pinniped playbacks as a non-threatening control. When integrated across the entire month-long treatment period, the magnitude of the effect of large carnivore-induced fear on crab abundance was even greater than when crabs were only sampled at the end of each treatment period – the mean (± s.e.m.) difference in the number of crabs per trap between predator and non-predator treatments was 3.9 (± 1.1) crabs (Supplementary Fig. 1)

Effects of fear of large carnivores on species not directly eaten by raccoons Given the breadth of raccoon impacts across the nearshore marine community[1] and the ability of fear-induced behavioural changes to mitigate these impacts (Fig. 3), we hypothesized that the indirect effects of large carnivore-induced fear may extend beyond those species directly subject to raccoon predation. In addition to being a common food source for raccoons, red rock crabs are themselves major intertidal predators, and may interact strongly with other intertidal predators of similar body size, including the staghorn sculpin (*Leptocottus armatus*), the most common sculpin on the Pacific Coast of North America[2]. Diets of red rock crabs and staghorn sculpins overlap considerably, with both species preying on a range of arthropods, bivalves and gastropods, and scavenging dead fish[2-8]. Red rock crab and staghorn sculpin habitat use also overlaps substantially[2,5], suggesting the potential for strong competition between these two intertidal predators. However, unlike red rock crabs, we have no evidence that staghorn sculpins are preyed upon by raccoons. Staghorn sculpins are comparatively large (c. 15 cm), free-swimming, fast-moving, fish[2]. Based on our many hundreds of hours recording raccoon foraging, raccoon predation on fish is limited to smaller species that remain in the intertidal at low tide, hidden under rocks (e.g., prickleback and northern clingfish), and we have never encountered staghorn sculpins under rocks at low tide during intertidal quadrat sampling (JPS, pers. obs.). Indeed staghorn sculpins may even benefit from raccoon presence due to increased scavenging opportunities, as raccoons regularly leave the carcasses of freshly killed crabs in the intertidal[1].

As intertidal predators, red rock crabs affect the abundance of several species of gastropod prey[4,9], including periwinkle snails[3,10-12]. Periwinkle snails are in turn major intertidal grazers[13,14] with the ability to control algal cover and diversity[15-17], particularly on sheltered coastlines[14] like those studied here. The local periwinkle species (*Littorina scutulata* and *L. sitkana*) are among the most abundant grazers along coastlines in and near the Gulf Islands ([3]; JPS, pers. obs.), and red rock crabs are likely their most important predators in many areas[10,18]. The strength of red rock crab predation on periwinkle snails therefore likely has significant cascading effects on the diversity and abundance of primary producers in this region.

Supplementary References

1. Suraci, J. P., Clinchy, M., Zanette, L. Y., Currie, C. M. A. & Dill, L. M. Mammalian mesopredators on islands directly impact both terrestrial and marine communities. *Oecologia* **176**, 1087 (2014).
2. Tasto, R. N. Aspects of the biology of Pacific staghorn sculpin, *Leptocottus armatus* Girard, in Anaheim Bay in *The Marine Resources of Anaheim Bay*. Eds. Lane, E.D. & Hill, C.W. (Fisheries Bulletin, 1975).
3. Behrens Yamada, S. & Boulding, E. G. The role of highly mobile crab predators in the intertidal zonation of their gastropod prey. *J. Exp. Mar. Biol. Ecol.* **204**, 59 (1996).
4. Robles, C., Sweetnam, D.A. & Dittman, D. Diel variation of intertidal foraging by *Cancer productus* L. in British Columbia. *J. Nat. Hist*. **23**, 1041 (1989).

5. Carroll, J. C. & Winn, R. N. Brown rock crab, red rock crab, and yellow crab. *Species profiles: Life histories and environmental requirements of coastal fishes and invertebrates (Pacific Southwest)* (United States Fish and Wildlife Service, 1989).
6. Armstrong, J. L., Armstrong, D. A. & Mathews, S. B. Food habits of estuarine staghorn sculpin, *Leptocottus armatus*, with focus on consumption of juvenile Dungeness crab, *Cancer magister*. *Oceanographic Literature Review* **6**, 595 (1996).
7. Smith, T. E., Ydenberg, R. C. & Elner, R. W. Foraging behaviour of an excavating predator, the red rock crab (*Cancer productus* Randall) on soft-shell clam (*Mya arenaria* L.). *J. Exp. Mar. Biol. Ecol*. **238**, 185 (1999).
8. Meyer, J. J. & Byers, J. E. As good as dead? Sublethal predation facilitates lethal predation on an intertidal clam. *Ecol. Lett*. **8**, 160 (2005).
9. Bourdeau, P. E. Morphological defense influences absolute, not relative, nonconsumptive effects in marine snails. *Behav. Ecol.* **24**, 505 (2013).
10. Rochette, R. & Dill, L. M. Mortality, behavior and the effects of predators on the intertidal distribution of littorinid gastropods. *J. Exp. Mar. Biol. Ecol*. **253**, 165 (2000).
11. McCormack, S. M. D. The maintenance of shore-level size gradients in an intertidal snail (*Littorina sitkana). Oecologia* **54**, 177 (1982).
12. Behrens Yamada, S., Navarrete, S. A. & Needham, C. Predation induced changes in behavior and growth rate in three populations of the intertidal snail, *Littorina sitkana* (Philippi). *J. Exp. Mar. Biol. Ecol*. **220**, 213 (1998).
13. Mrowicki, R. J. & O'Connor, N. E. Wave action modifies the effects of consumer diversity and warming on algal assemblages. *Ecology* **96**, 1020 (2015).
14. Lindegarth, P. A. M. Effects of grazing on the structure of mid-shore, intertidal assemblages on moderately exposed rocky shores of the Swedish west coast. *Mar. Ecol. Prog. Ser*. **212**, 29 (2001).
15. Bertness, M. D., Yund, P. O. & Brown, A. F. Snail grazing and the abundance of algal crusts on a sheltered New England rocky beach. *J. Exp. Mar. Biol. Ecol*. **71**, 147 (1983).
16. Lubchenco, J. Plant species diversity in a marine intertidal community: Importance of herbivore food preference and algal competitive abilities. *Am. Nat*. **112**, 23 (1978).
17. Lubchenco, J. Littornia and Fucus: Effects of herbivores, substratum heterogeneity, and plant escapes during succession. *Ecology*. **64**, 1116 (1983).
18. Boulding, E. G., Holst, M. & Pilon, V. Changes in selection on gastropod shell size and thickness with wave-exposure on northeastern Pacific shores. *J. Exp. Mar. Biol. Ecol*. **232**, 217 (1999).
